# BeiDou Signal Acquisition with Neumann–Hoffman Code Modulation in a Degraded Channel

**DOI:** 10.3390/s17020323

**Published:** 2017-02-09

**Authors:** Lin Zhao, Aimeng Liu, Jicheng Ding, Jing Wang

**Affiliations:** Harbin Engineering University, College of Automation, Harbin 150001, China; zhaolin@hrbeu.edu.cn (L.Z.); aimengliu@163.com (A.L.); wangjing19900226@hrbeu.edu.cn (J.W.)

**Keywords:** BeiDou, acquisition, bit transition, differential coherent integration

## Abstract

With the modernization of global navigation satellite systems (GNSS), secondary codes, also known as the Neumann–Hoffman (NH) codes, are modulated on the satellite signal to obtain a better positioning performance. However, this leads to an attenuation of the acquisition sensitivity of classic integration algorithms because of the frequent bit transitions that refer to the NH codes. Taking weak BeiDou navigation satellite system (BDS) signals as objects, the present study analyzes the side effect of NH codes on acquisition in detail and derives a straightforward formula, which indicates that bit transitions decrease the frequency accuracy. To meet the requirement of carrier-tracking loop initialization, a frequency recalculation algorithm is proposed based on verified fast Fourier transform (FFT) to mitigate the effect, meanwhile, the starting point of NH codes is found. Then, a differential correction is utilized to improve the acquisition accuracy of code phase. Monte Carlo simulations and real BDS data tests demonstrate that the new structure is superior to the conventional algorithms both in detection probability and frequency accuracy in a degraded channel.

## 1. Introduction

The application of global navigation satellite systems (GNSS) in harsh environments is drawing increasing attention among civilian users [1]. As one of the world’s major navigation system, BDS is scheduled to be completed in 2020, and will eventually operate in ocean, urban, and indoor environments. To improve the capability for anti-narrowband interference and provide more precise ranging information, a secondary modulation of the NH code is introduced [2,3]. However, the periodicity of the transmitted sequence was broken by the NH codes, leading to more polarity changes, also known as bit transitions.

For typical receiver modules, the acquisition process occurs first and determines the sensitivity of a receiver [4]. The maximum sensitivity gain is achievable by the integration of consecutive correlations of the received signal and the local code replica [5]. The integration time becomes one of the main concerns that should be addressed in challenging environments affected by bit transitions, square loss, computational burden, and so on [6]. 

Coherent combination (CC) is the most classical signal acquisition algorithm which can obtain the maximum signal-to-noise ratio (SNR), but the integration time is limited by bit transitions [7]. In order to solve this problem, some improved CC algorithms are proposed. The half-bit alternation method that combines CC can be adopted to skip bit transitions [8], and a block acquisition scheme using the CC method can also alleviate this effect [9], meanwhile, a number of weighted signal blocks can be coherently accumulated to obtain an improved power [10]. However, these algorithms based on the foundation that the probability of bit transitions is 5% for the global positioning system (GPS) L1 signal. However, the probability of bit transitions is 50% for the BDS B1 signal caused by the NH codes, which can significantly influence the aforementioned integration algorithms [11,12].

Apart from the CC method and its modified version, two other well-known techniques, namely, the non-coherent combination (NC) algorithm and the differential combination (DC) algorithm, are also applied in weak signal environments [13,14]. The NC algorithm mitigates the undesirable effect of polarity changes through a squaring operation, but has to suffer from square loss, which reduces the SNR [15,16]. The DC method is notable for multiplying the present integration result with the delay conjugate of the last one, which reduces the square loss and the effect of polarity changes to a certain extent, thereby making this method feasible in weak GPS signal processing [17]. Different forms of detection schemes can be found in literature [17,18,19].

Regardless of the calculation burden, bit transition is a major problem that hinders long integration time, it can degrade the probability of acquisition in terms of Galileo E1 OS, GPS L5, and GPS L1C signal in different degrees [20], as well as the BDS B1 signal with a similar modulation. To achieve high frequency accuracy and acquisition probability in weak environments, an aiding Doppler information from strap-down inertial navigation system can be added into the acquisition process to reduce the search band of frequency. Meanwhile, the Viterbi algorithm is used for bit estimation to obtain a long differential integration time [6]. To demodulate the NH code without aiding information, a search tree for secondary codes is proposed combined with the CC method [21]; however, 2^20^ combinations for 20 ms integration time will consume a very large amount of storage. Simply adopting a whole tiered code of pseudo-random noise (PRN) codes and NH codes as the local code for acquisition is an easier alternative [22], however, conducting that many FFTs and inverse FFTs of a 20 ms sequence in each frequency unit is difficult. On the basis of the same principle, a modified CC method, that uses the top 5 ms NH chips with the same polarity as the local code, is utilized to acquire BDS signal [23], but this technique requires 19 instances of redundant computation in each frequency unit.

In the above algorithms, FFT is used for transformation from the time domain to the frequency domain, or from the frequency domain to the time domain, to reduce the computation burden. Additionally, the basic function of FFT is to analyze the spectrum with a simplified search method, and the resolution of the spectrum is proportional to the length of the data [24]. Based on the theory and the analysis of paper [25], the present study derives a formula that shows exactly how bit transitions can bring in an error Doppler estimation, and related analysis is addressed. A frequency recalculation algorithm is proposed which exploits the efficiency of FFT-based algorithms, the start point of NH codes can be found, that is to say the symbol combination is found, and long integration time becomes possible. Meanwhile, a more accurate Doppler bins can be estimated. This result is helpful for speeding up the tracking process. The developed algorithm is analyzed in terms of cross-ambiguity function (CAF). Monte Carlo simulations are used to analysis the results, and the algorithms are demonstrated through real data. 

The remainder of this paper is organized as follows: Section 2 introduces the BDS signal model and reviews the acquisition process. Section 3 analyzes the side effect of bit transitions on acquisition and derives a theoretical formula in the presence of bit transitions. In Section 4, a FFT-based acquisition algorithm is proposed to get the fine frequency. In Section 5 the different integration techniques are further analyzed and compared by means of Monte Carlo simulations and real data. Finally, conclusions are drawn in Section 6.

## 2. Signal and System Model 

**Figure 1:**
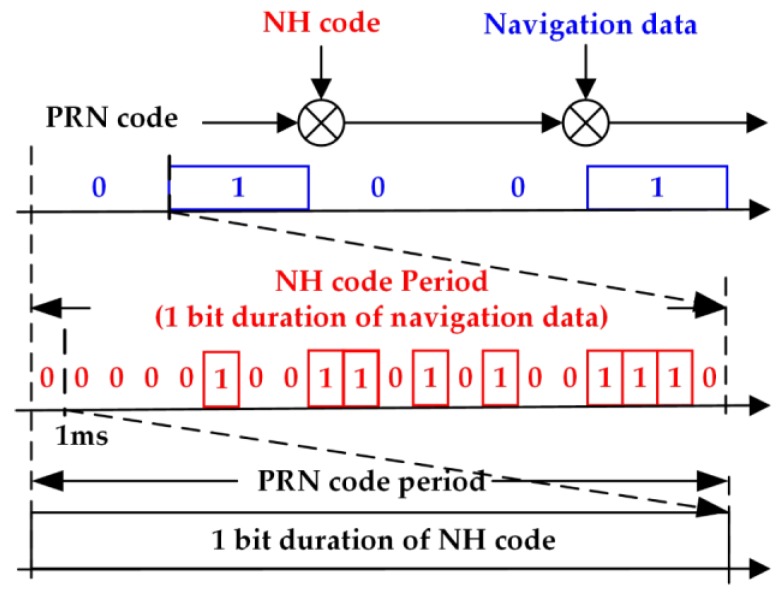
BDS signal adopting NH code modulation

Unlike conventional GPS signals, BDS B1 signal adopts secondary codes modulation by the primary purpose of speeding up bit synchronization. Thus, the period of NH codes is 20 ms, corresponding to a navigation bit interval, besides, the secondary codes and the navigation bits are time aligned. Figure 1 shows the structure of BDS B1 signal. The received signal of the B1 in-phase branch in a one-path additive white Gaussian noise channel can be modeled as [26]:

(1) $$y\left(t\right)=\sum_{k=1}^{K}A^{k}d^{k}\left(t-\tau_{0}^{k}\right)c^{k}\left(t-\tau_{0}^{k}\right)s^{k}\left(t-\tau_{0}^{k}\right)\cdot \cos\left(2\pi \left(f_{\mathrm{RF}}+f_{0}^{k}\right)t+\varphi_{0}^{k}\right)+\eta \left(t\right)$$

where, superscript *k* stands for satellite number depending on the modulated PRN code; *A* is the amplitude; and *K* is the number of visible satellites. *c*, *d*, and *s* represent the PRN codes, the navigation data and the NH codes, respectively. $f_{\mathrm{RF}}$ is the carrier frequency, while $f_{0}$, $\tau_{0}$, and $\varphi_{0}$ are the Doppler frequency, the delay and the carrier phase introduced when transmission. η is a stationary additive white Gaussian noise with variance $\sigma_{\eta }^{2}$.

The received signal is filtered, down-converted, sampled at the frequency $f_{s}=1/T_{s}$, and then digitalized. Thus, the digital signal can be expressed as:

(2) $$y\left[n\right]=\sum_{k=1}^{K}A^{k}d^{k}\left(nT_{s}-\tau_{0}^{k}\right)c^{k}\left(nT_{s}-\tau_{0}^{k}\right)s^{k}\left(nT_{s}-\tau_{0}^{k}\right)\cdot \cos\left(2\pi \left(f_{\mathrm{IF}}+f_{0}^{k}\right)nT_{s}+\varphi_{0}^{k}\right)+\eta \left(nT_{s}\right)$$

where $f_{\mathrm{IF}}$ is the receiver intermediate frequency.

Owing to the quasi-orthogonality of the PRN codes, different signals can be analyzed separately. Thus, the following signal model can be adopted for simplification:

(3) $$y\left[n\right]=Ad\left(nT_{s}-\tau_{0}\right)c\left(nT_{s}-\tau_{0}\right)s\left(nT_{s}-\tau_{0}\right)\cdot \cos\left(2\pi F_{0}n+\varphi_{0}\right)+\eta \left(nT_{s}\right)$$

where the index k has been dropped, and $F_{0}=\left(f_{\mathrm{IF}}+f_{0}\right)T_{s}$ is introduced. It should be noted that the input signal of conventional GPS L1 signal has a similar form, but there are no secondary codes, that is, there is no $s\left(nT_{s}-\tau_{0}\right)$ term.

The basic concept of signal acquisition is to obtain available satellite PRN numbers, estimated Doppler shifts, and code phases through correlation between signal of Equation (3) and the local duplicates, then a random variable $R\left(F_{D},\tau \right)$ is produced, where, $F_{D}=\left(f_{\mathrm{IF}}+f_{d}\right)T_{s}$ and τ stands for different carrier frequencies and delays to be tested. When the GPS L1 signal is present [26]:

(4) $$R\left(F_{D},\tau \right)=\frac{A}{2}d\frac{\sin\left(\pi N\Delta F\right)}{\pi N\Delta F}R\left(\Delta \tau \right)\exp\left(-j\Delta \varphi \right)+\eta$$

R(⋅) is the cross correlation between the input and the local code, N is number of samples, and $T_{c}=NT_{s}$ defines the coherent integration time in seconds, $T_{c}$ is set to 0.001s. $\Delta F=F_{0}-F_{D}$ is the frequency difference between the input and local signal, while $\Delta \tau =(\tau_{0}-\tau )/T_{s}$ represents the difference between the input and local code delay, normalized by sampling interval $T_{s}$, Δφ is the difference of phases between the input and local carriers, d is 1 or −1 that represents the navigation bits. Equation (4) is derived based on the assumption that the navigation bits will not reverse during the integration process. It is reasonable because there exists, at most, one bit transition in every 20 ms, though the one variable affected by bit transition is no longer in the same form with Equation (4), it hardly affect the final result. However, when it comes to the BDS B1 signal, the equation is not applicable, and the general Equation (4) must be revised attributing to the NH codes. The next section will derive a new variable that fits for the BDS B1 signal and a detailed analysis will be drawn. 

## 3. Analysis of Bit Transitions

The CC acquisition is realized by averaging the in-phase (I) and quadrature (Q) components through correlation [27]. The derivation of variable $R\left(F_{D},\tau \right)$ can easily be found in the related literature, thus, part of the derivation in this paper is omitted. The same goes for the derivation of decision variable for GPS signals, the assumption that navigation bits will not reverse during the integration process is made. When the local code replica perfectly matches the input signal, that is $\tau =\tau_{0}$, the CC results can be expressed as:

(5) $$\begin{array}{ll} R\left(F_{D},\tau \right) & =\sum_{n=0}^{N-1}Ad\left(nT_{s}-\tau_{0}\right)c\left(nT_{s}-\tau_{0}\right)s\left(nT_{s}-\tau_{0}\right)\cos\left(2\pi F_{0}n+\varphi_{0}\right)\\ & \;\;\cdot c_{loc}\left(nT_{s}-\tau \right)\exp\left(j2\pi F_{D}n\right)+\eta \\ & =\frac{A}{2}\sum_{n=0}^{N-1}s\left(nT_{s}-\tau_{0}\right)\cdot \{\exp\left[-j\left(2\pi \Delta Fn+\varphi \right)\right]+\exp\left[j\left(2\pi \left(F_{0}+F_{D}\right)n+\varphi \right)\right]\}+\eta \end{array}$$

Suppose that bit transitions occur in $N_{\tau }$ ($0\leq N_{\tau }\leq N-1$), namely:

(6) $$s\left(nT_{s}-\tau_{0}\right)=\{\begin{array}{ccc} 1 & for & 0\leq n<N_{\tau }\\ -1 & for & N_{\tau }\leq n\leq N-1 \end{array}$$

Considering that high frequency components can be mitigated by integration, then,

(7) $$R\left(F_{D},\tau \right)\approx \frac{A}{2}\exp\left(-j\varphi \right)\left\{\sum_{n=0}^{N_{\tau }-1}\exp\left[-j\left(2\pi \Delta Fn\right)\right]-\sum_{n=N_{\tau }}^{N-1}\exp\left[-j\left(2\pi \Delta Fn\right)\right]\right\}+\eta$$

The two terms in Equation (7) are two truncated geometrical series, which can be summed easily as [25]:

(8) $$\begin{array}{ll} R\left(F_{D},\tau \right) & \approx \frac{A}{2}\exp\left(-j\varphi \right)\sum_{n=0}^{N-1}s\left(nT_{s}-\tau_{0}\right)\exp\left(-j2\pi \Delta Fn\right)\\ & =\frac{A}{2}\exp\left(-j\varphi \right)\cdot \{\exp\left(j\alpha_{1}\right)\frac{\sin\left(\pi \Delta FN_{\tau }\right)}{\sin\left(\pi \Delta F\right)}-\exp\left(j\alpha_{2}\right)\frac{\sin\left[\pi \Delta F\left(N-N_{\tau }\right)\right]}{\sin\left(\pi \Delta F\right)}\} \end{array}$$

where $\alpha_{1}=\pi \Delta F\left(N_{\tau }-1\right)$ and $\alpha_{2}=\pi \Delta F\left(N+N_{\tau }-1\right)$. When $N_{\tau }=N/2$, $R\left(F_{D},\tau \right)$ can be rewritten as:

(9) $$\begin{array}{ll} R\left(F_{D},\tau \right) & =\frac{A}{2}\exp\left(-j\varphi \right)\{\exp\left[j\pi \Delta F\left(\frac{N}{2}-1\right)\right]-\exp\left[j\pi \Delta F\left(\frac{3N}{2}-1\right)\right]\}\frac{\sin\left(\frac{1}{2}\pi \Delta FN\right)}{\sin\left(\pi \Delta F\right)}\\ & =\frac{A}{2}\exp\left(-j\varphi \right)\exp\left[j\pi \Delta F\left(\frac{N}{2}-1\right)\right]\cdot \left[1-\exp\left(j\pi \Delta FN\right)\right]\Phi \left(\Delta F\right)\\ & =\frac{A}{2}\exp\left(-j\varphi \right)\Gamma \left(\Delta F\right)\Phi \left(\Delta F\right) \end{array}$$

where, $\Phi \left(\Delta F\right)=\sin\left(\frac{1}{2}\pi \Delta FN\right)/\sin\left(\pi \Delta F\right)$, and $\Gamma \left(\Delta F\right)=\exp\left[j\pi \Delta F\left(\frac{N}{2}-1\right)\right]\cdot \left[1-\exp\left(j\pi \Delta FN\right)\right]$.

The Doppler frequency is within 10 kHz under the existing vehicle and satellite conditions [28], whereas $f_{s}$ is greater than twice the intermediate frequency (IF). That is, ΔF, which denotes the ratio of the frequency error, and $f_{s}$, is extremely small, i.e., smaller than 0.0025. Thus, sin(πΔF) can be replaced with πΔF as follows:

(10) $$\begin{array}{ll} R\left(F_{D},\tau \right) & =\frac{AN}{4}\exp\left(-j\varphi \right)\frac{\sin\left(\pi \Delta FN/2\right)}{\pi \Delta FN/2}\Gamma \left(\Delta F\right)\\ & =\frac{AN}{4}\exp\left(-j\varphi \right)\mathrm{sinc}\left(\Delta FN/2\right)\Gamma \left(\Delta F\right) \end{array}$$

Then, the decision variable of CC is determined as:

(11) $$\begin{array}{ll} S\left(F_{D},\tau \right) & ={\left|R\left(F_{D},\tau \right)\right|}^{2}={\left[\frac{AN}{4}\mathrm{sinc}\left(\frac{1}{2}\Delta FN\right)\right]}^{2}{\left|1-\exp\left(j\pi \Delta FN\right)\right|}^{2}\\ & \;=2{\left[\frac{AN}{4}\mathrm{sinc}\left(\frac{1}{2}\Delta FN\right)\right]}^{2}\left[1-\cos\left(\pi \Delta FN\right)\right] \end{array}$$

The NC technique is realized by summing the squared coherent correlation values. This method can be written as [13]:

(12) $$N\left(F_{D},\tau \right)=\sum_{n=1}^{N_{\mathrm{NC}}}{\left|R_{n}\left(F_{D},\tau \right)\right|}^{2}$$

where $N_{\mathrm{NC}}$ is the time of non-coherent integration. However, a famous square loss will be experienced because the noise is also squared [15].

The DC method will be achieved via two consecutive coherent integration periods. The output can be presented as follows [19]:

(13) $$Y\left(F_{D},\tau \right)=\left|\sum_{n=1}^{N_{\mathrm{DC}}}R_{n}\left(F_{D},\tau \right)\cdot R_{n-1}{\left(F_{D},\tau \right)}^{*}\right|$$

where $N_{\mathrm{DC}}$ is the time of differential integration; $R_{n}\left(F_{D},\tau \right)$ and $R_{n-1}{\left(F_{D},\tau \right)}^{*}$ are the *n*th output and the complex conjugate of the (*n*−1)-th output, respectively.

**Figure 2:**
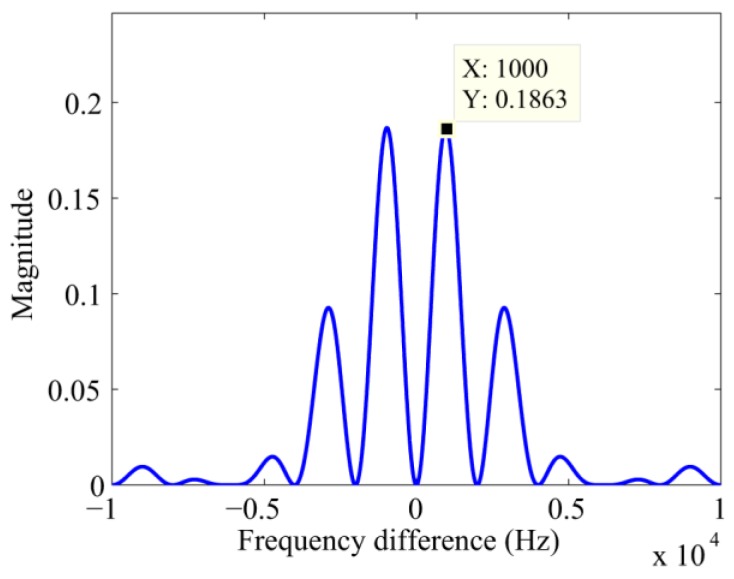
The curve of ${\mathrm{sinc}}^{2}\left(\Delta FN/2\right)\left[1-\cos\left(\pi \Delta FN\right)\right]$.

In Equation (11), ${\mathrm{sinc}}^{2}\left(\Delta FN/2\right)$ and [1−cos(πΔFN)] will determine the position of the peak in the frequency bins. ${\mathrm{sinc}}^{2}\left(\Delta FN/2\right)$ will monotonically decrease to zero in |ΔF|∈[0,2/N], in other words, the peak decreases as the frequency error increases in the range $\left|\Delta f\right|=\left|\Delta F\right|\cdot f_{s}\in \left[0,2000\right]$ Hz. However, when ΔF is equal to zero, [1−cos(πΔFN)] is equal to zero too. That is to say, the peak is no longer consistent with the right Doppler bins. When ΔF is equal to ±1/N, i.e., the frequency difference is equal to ±1000 Hz, a maximum of [1−cos(πΔFN)] will be achieved and ${\mathrm{sinc}}^{2}\left(\Delta FN/2\right)\left[1-\cos\left(\pi \Delta FN\right)\right]$ will reach its maximum, which will bring in a large frequency error. The curve of ${\mathrm{sinc}}^{2}\left(\Delta FN/2\right)\left[1-\cos\left(\pi \Delta FN\right)\right]$ is illustrated in Figure 2. 

**Figure 3:**
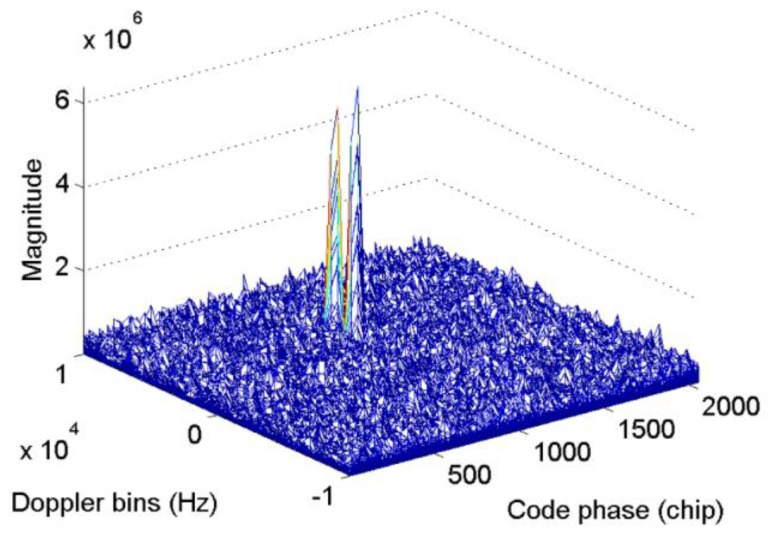
Acquisition results with simulated signal of satellite 12.

Figure 3 presents the acquisition results with simulated BDS signal of satellite 12 when $T_{c}=0.001$s, $f_{s}=16.368$ MHz, and $f_{\mathrm{IF}}=4.092$ MHz, thus, N=1,6368, the code phase, and also $N_{\tau }$, is set as 0.5N. The starting point of the NH codes are set to 11 NH code chips, and the Doppler bins are set to 3000 Hz. The bin width of the frequency dimension and code dimension are 500 Hz and 0.5 chips, respectively, for the full paper. As shown in Figure 3, when the integration process crosses bit transitions, the peak in the frequency axis splits into two components. The corresponding Doppler bins of the peak is 2500 Hz, which indicates a failed acquisition. Considering that the CC method serves as the base of the NC and DC methods, the outputs of CC are accumulated by means of square or delay conjugate multiplication to reduce the white Gaussian noise. However, such operations cannot eliminate the frequency error, and it will be transmitted to the final decision variable. Moreover, Equation (11) indicates that the code phase is still correct, except for a loss of amplitude. Consequently, a FFT-based frequency recalculation method is designed to mitigate the undesirable effect of bit transitions.

## 4. FFT Based Frequency Recalculation

Considering that the frequency of conventional acquisition algorithms deviates from the desired value because of the secondary codes, a more accurate frequency must be obtained. Generally, the frequency spectrum of a digital sine wave can be obtained via discrete Fourier transform, and the revolution of the spectrum is proportional to the ratio of the sampling frequency and the samples. To apply the theory to GNSS signal acquisition, the PRN codes, NH codes, and navigation bits must be demodulated from the received signal to obtain the spectrum of the carrier. The code phase estimated from conventional acquisition algorithms can be used to remove the PRN code. Thus, there remains the NH codes and navigation bits.

**Figure 4:**
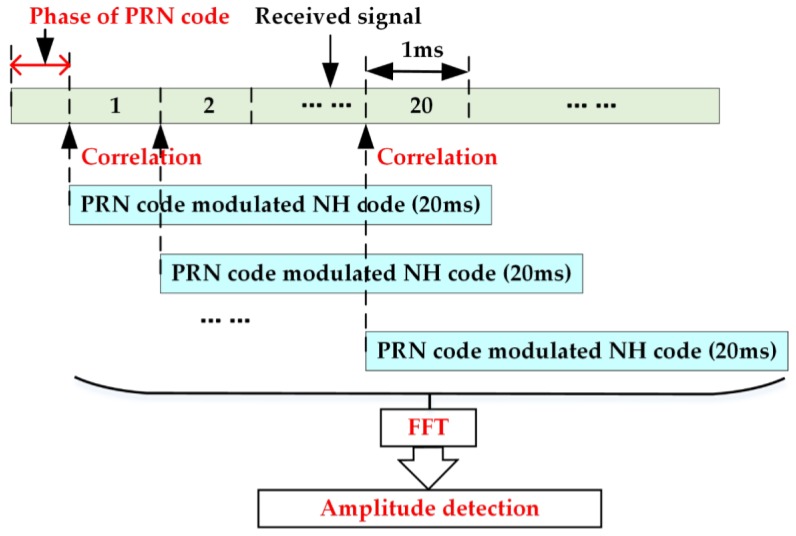
Diagram of the frequency recalculation algorithm.

In this study, a frequency search structure is adopted to remove the NH codes and the navigation bits. First, we conduct a conventional CC acquisition to obtain the coarse code phase of PRN codes. Then, we make the local PRN codes align with the received signal at the first bit edge. Next, wew correlate the signal of step two with the whole tiered codes of the PRN codes and NH codes 20 times, of which the delay is 1 ms. Finally, we convert the correlations into frequency domain. There will be 20 outputs, and each output has a maximum value. The largest of these maximums means that both the NH codes and the navigation bits are removed, and a more precise frequency of the received signal is obtained. The diagram of the frequency search structure is shown in Figure 4. 

In open areas, the length of the FFT can be shortened. More specifically, any consecutive chips of the PRN-NH modulated codes can be used for the proposed algorithm. However, the sensitivity differs significantly, as shown in the next section. To satisfy the requirement of the tracking loop initialization in weak signal environments, the whole tiered codes of the PRN and NH codes are a better choice. 

**Figure 5:**
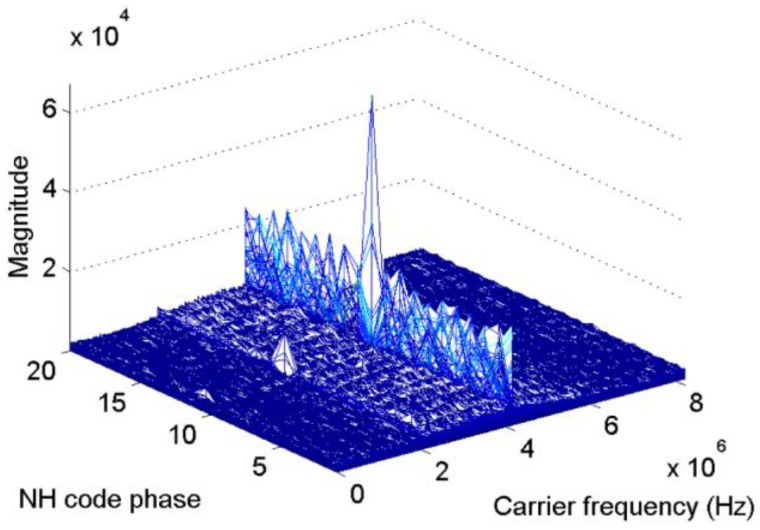
Frequency recalculation result against the NH code phase and carrier frequency.

**Figure 6:**
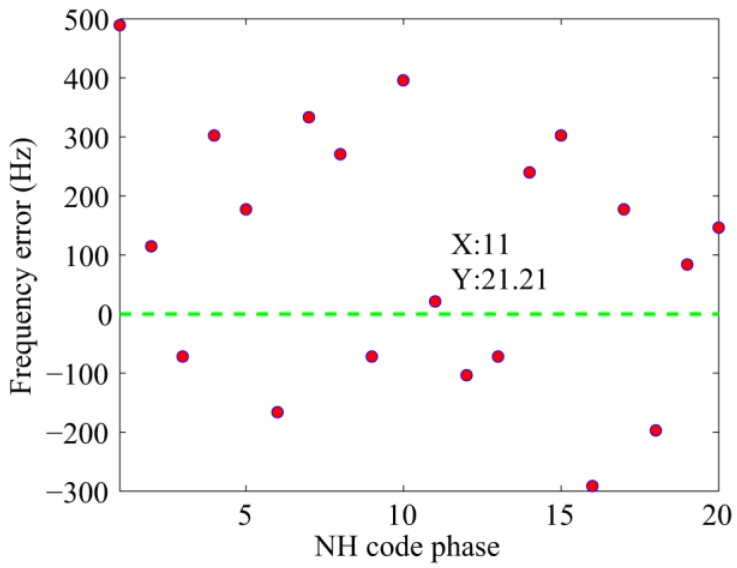
Frequency errors against different NH code phases.

Figure 5 and Figure 6 present the results of the new scheme of the same signal used in Figure 3. These parameters will not repeat here. In Figure 5, the length of the FFT is 524,288 samples, That is, the revolution of the spectrum is $f_{s}/524288=31.2$ Hz. Figure 5 shows that the index of the maximum value is 11, which is consistent with the default value. Meanwhile, in the carrier frequency dimension, the peak appears at 4,095,018 Hz, which represents a slight error of 18 Hz. Figure 6 shows the frequency error against different NH code phases in a more direct way. Only when the local NH codes is aligned with the received signal can the peak reach the maximum value. In other cases, the frequency error of tens to hundreds of hertz occurs. 

When frequent bit transitions are considered in urban environments, the NC algorithm should be adopted before using the FFT-based frequency recalculation algorithm to increase the peak. In addition to the accuracy frequency, the start point of NH codes can also be found through the proposed scheme, thus, the NH codes can be removed, and there remains only the navigation bits. Then, a conventional DC algorithm will easily be conducted with the fine frequency to correct the code phase of the DC algorithm. The final code phase $\bar{\tau }$ of the scheme is determined by the following criterion:

(14) $$\{\begin{array}{ll} \bar{\tau }=\tau_{\mathrm{NC}} & for\;r_{\mathrm{NC}}>r_{\mathrm{DC}}\\ \bar{\tau }=\tau_{\mathrm{NC}}-\Delta \tau_{\mathrm{DC}} & for\;r_{\mathrm{NC}}\leq r_{\mathrm{DC}} \end{array}$$

where, $r_{\mathrm{NC}}$ and $r_{\mathrm{DC}}$ represent the peak to second noise ratio of the NC and DC algorithms, respectively. The peak to second noise ratio is usually used for threshold detection, here, it indicates the reliability of the conventional NC and DC algorithms as well. The outputs with high peak to second noise ratio are more reliable.$\tau_{\mathrm{NC}}$ and $\Delta \tau_{\mathrm{DC}}$ denote the code phases obtained from the conventional NC and DC algorithms, respectively. More specifically, $\Delta \tau_{\mathrm{DC}}$ stands for the code phase error of the NC algorithm owing to step 2 of the frequency recalculation algorithm. If the code phase estimation $\tau_{\mathrm{NC}}$ is correct, $\Delta \tau_{\mathrm{DC}}$ equals 0; if not, $\tau_{\mathrm{NC}}$ is corrected with $\Delta \tau_{\mathrm{DC}}$.

## 5. Performance Assessment

To achieve a comprehensive assessment of the proposed acquisition scheme, Monte Carlo simulations were conducted to compare the proposed scheme with the conventional NC and DC algorithms. A schematic simulation platform was designed. Furthermore, BDS signals received by a GNSS receiver and other auxiliary equipment were also used to implement the expected scheme.

### 5.1. Monte Carlo Simulations

Monte Carlo simulations were used for comprehensive evaluation of different algorithms. To prove the necessity and reliability of the proposed algorithm, comparisons were done to study the sensitivity of different signal lengths, and the influence of the coarse code phase obtained from the conventional NC algorithm was analyzed. Then, the detection probability and frequency error were plotted as a function of the C/N_0_s and the bit transition positions for comparison with the conventional algorithms. 

**Figure 7:**
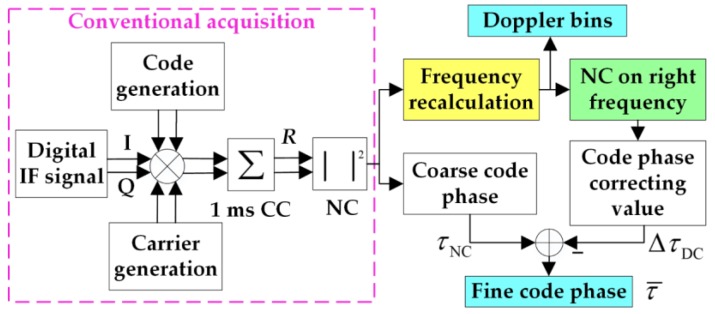
The diagram of the simulation platform.

The platform is shown in Figure 7, and the signal channel could be achieved by adjusting the amplitude and delay of the IF signal. The following basic settings were used to obtain statistic results: A software simulator generates the BDS signal with an intermediate frequency of 4.092 MHz and a sampling frequency of 16.368 MHz. The Doppler frequency distributes uniformly in [−10000, +10000] Hz, and the code phase distributes uniformly in [0, 1] chips. The front-end filter bandwidth is 4.5 MHz. The coherent integration time was set to 1 ms, considering the secondary code. Furthermore, additional white Gaussian noise was generated for each trial, and 1000 trials were used for each probability and frequency value.

**Figure 8:**
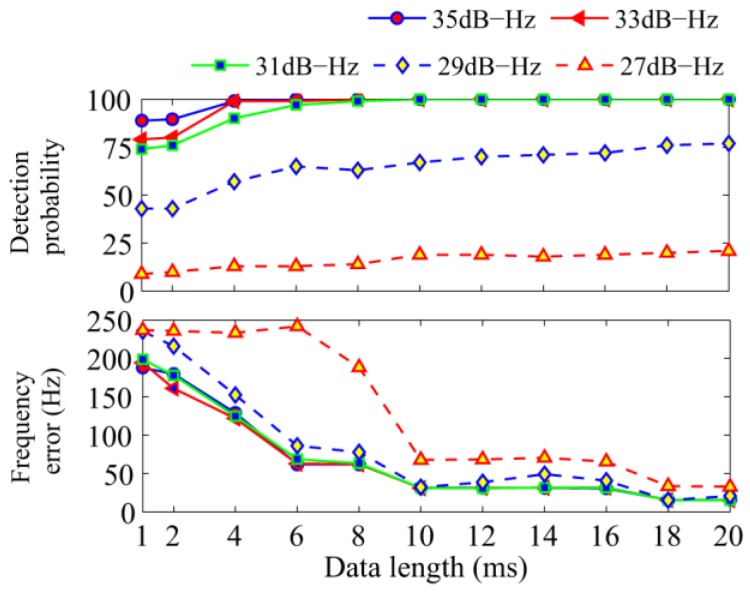
Influence of the FFT length on the proposed algorithm.

The first simulation tests the effect of signal lengths on the proposed algorithm. The total integration time was set to 100 ms, and different C/N_0_s were tested. The detection probability and average frequency error are shown in Figure 8. With the increase of the length of FFT, the detection probability increases as well, while the frequency error decreases. It is clear that, with the increase of C/N_0,_ the performance improves greatly. When the C/N_0_ is greater than 31 dB-Hz and the data length is greater than 8 ms, the performance differs slightly only in the error of the frequency. However, different data lengths show a large difference in the frequency error. In high C/N_0_ conditions, the FFT of 10ms length or shorter is enough for the tracking process. Considering the conditions in the following analysis, 20 ms-length FFT is used for better performance if there is no special notation.

**Figure 9:**
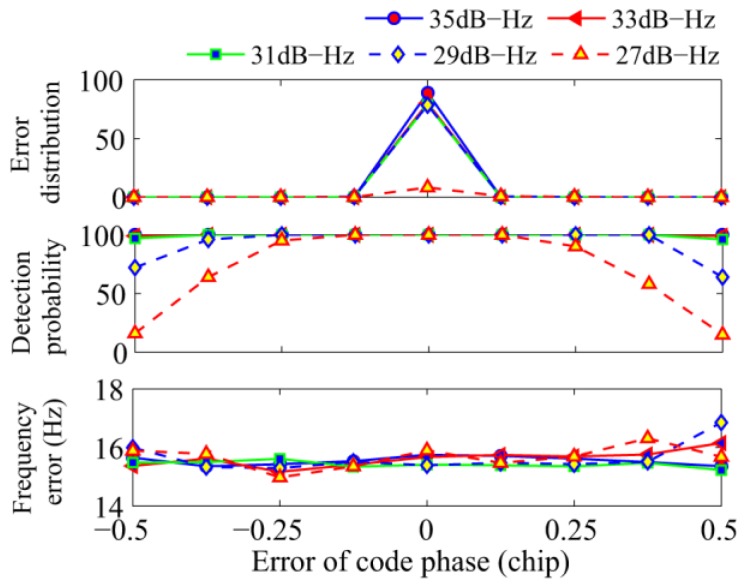
Performance of the proposed algorithm against the code phase error.

The second simulation analyzes the reliability of the proposed algorithm against the accuracy of the coarse code phase. The total integration time was set to 100 ms. The error of code phase obtained from the conventional NC algorithm was set to [−0.5, +0.5] PRN code chips, which are equivalent to [−4, +4] samples. The results are shown in Figure 9. The top panel shows the distribution of the code phase error of successful NC acquisition, and the code phase error have been rounded up and down to an integral sample. It is clear that the error of the code phase distributes mainly within one sample. The middle and bottom panels show the detection probability and frequency error of the proposed algorithm, respectively. When the error of code phase is less than or equal to 0.125 chips, there is no loss of detection probability. When the code phase error increases further, the detection probability decreases. While the frequency error of the proposed algorithm remains stable.

**Figure 10:**
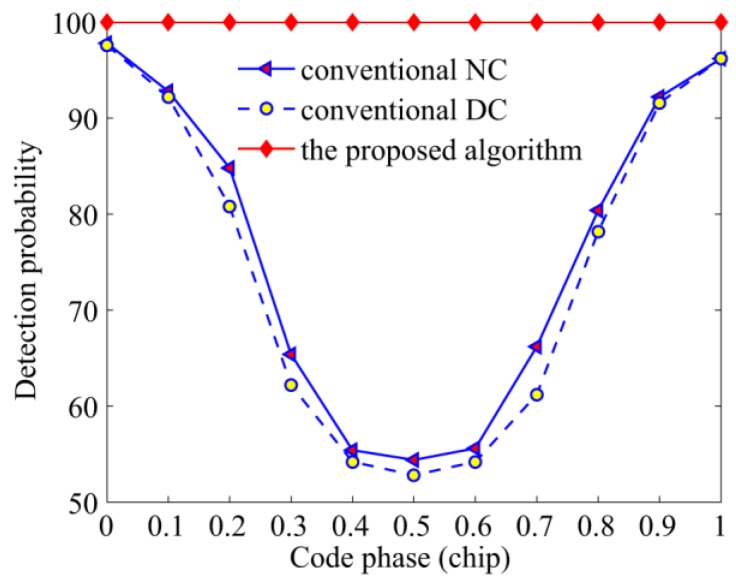
Detection probabilities of the NC, DC, and the proposed algorithms against the code phase.

**Figure 11:**
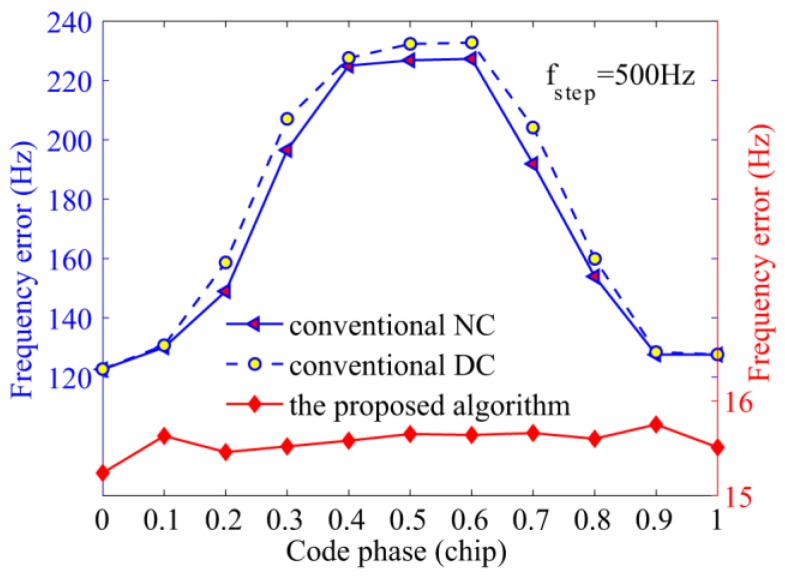
Average frequency errors of the NC, DC, and the proposed algorithms against the code phase.

The third simulation verifies a comparable acquisition performance among the three algorithms against the position of bit transitions. To better observe the side effect of bit transitions, the total integration time was set to 10 ms, and a high C/N_0_ of 45 dB-Hz was adopted. The detection probability and average frequency error are shown in Figure 10 and Figure 11, respectively, which clearly indicate that the detection probability and frequency error of the conventional NC and DC algorithms are functions of bit transition positions. More specifically, there shows a valley in the curve of detection probability when bit transitions appear on 0.5 chips, and the maximums show up on both sides while the frequency error presents the opposite trend. This phenomenon is consistent with the analysis of Section 3 that bit transitions do affect the acquisition performance. As for the reason why the performance of the NC algorithm is better than that of the DC algorithm, the next paragraph will give a detailed analysis. In contrast, the detection probability of the proposed algorithm is hardly affected by bit transitions, and the frequency error is much smaller than the conventional algorithms.

**Figure 12:**
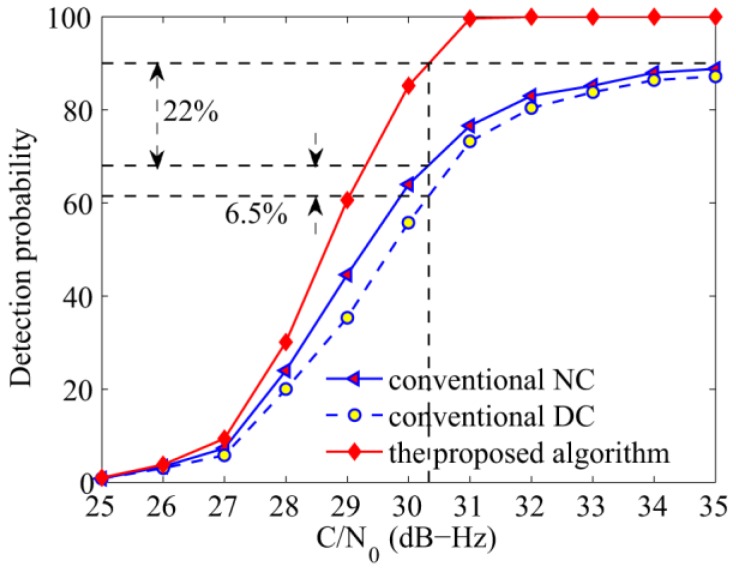
Detection probabilities of the NC, DC, and the proposed algorithms against C/N_0_.

**Figure 13:**
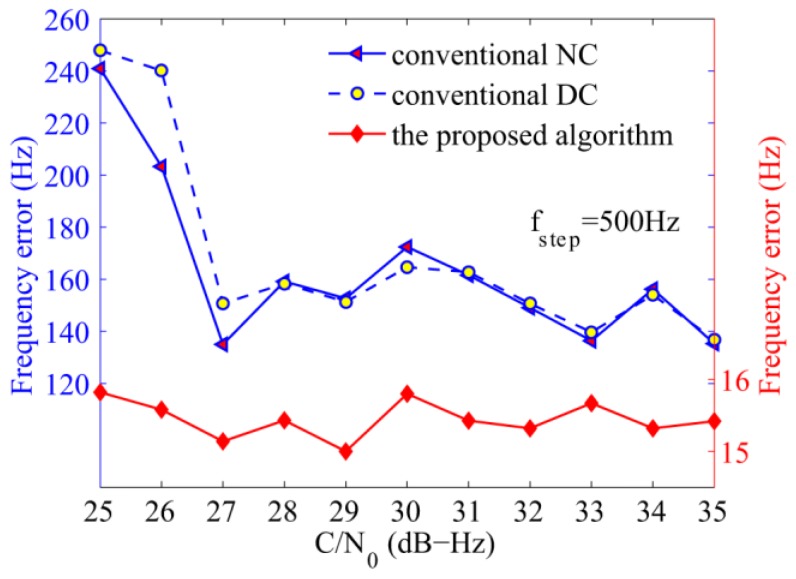
Average frequency errors of the NC, DC, and the proposed algorithms against C/N_0_.

The fourth simulation verifies the acquisition performance against C/N_0_ ranging from 25 dB-Hz to 35 dB-Hz, the total integration time was set to 100 ms. It is clear that the proposed algorithm demonstrates considerable advantage in Figure 12. When the C/N_0_ is 30.3 dB-Hz, the proposed algorithm can achieve a probability of 90%, which is 22% higher than that of the NC algorithm, and 6.5% more than the DC algorithm. As is known, when the GPS L1 signal is present, the bit transition loss of the DC algorithm can be negligible when long integration time is experienced, and the acquisition probability is higher than that of the NC algorithm. However, Figure 12 shows that the NC algorithm exhibits a slightly better performance than the DC algorithm although it has to endure squared losses, it is consistent with the analysis that frequent bit transitions of NH codes can deteriorate the acquisition performance, especially of algorithms that are affected by the polarity of the variable. Note that the DC algorithm is not affected directly by the signal polarity, but the product of two adjacent units. Considering the polarity of the secondary codes, bit transitions can decrease the performance of the DC algorithm, too. The analysis also applies to the results of the frequency in Figure 13. Moreover, it is shown that the detection probabilities of the NC and DC algorithms increase slowly when the C/N_0_ is higher than 32 dB-Hz, because the performance is mainly affected by frequent bit transitions in this case. Large frequency errors caused by bit transitions are the main reason, while C/N_0_ is only the secondary cause. 

### 5.2. Real Data Tests

**Figure 14:**
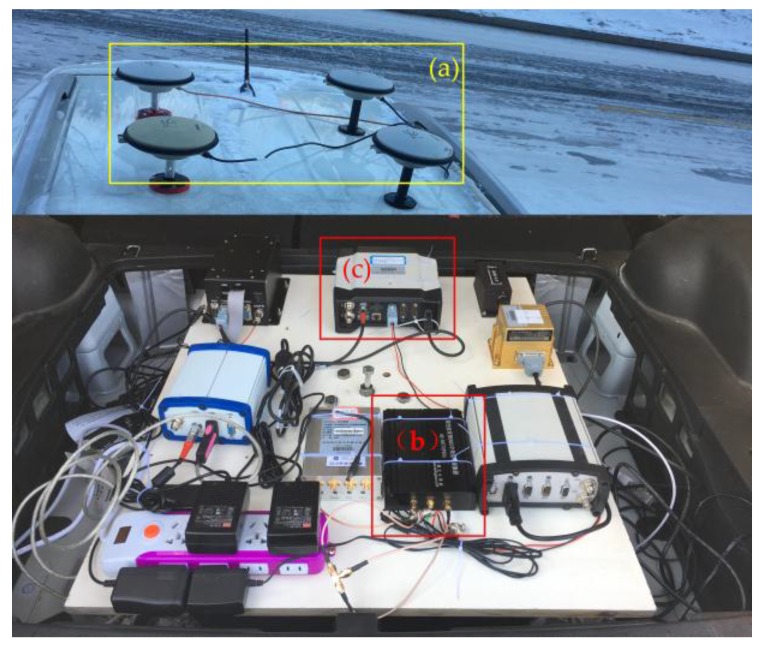
GNSS comprehensive test platform, (**a**) stand for GNSS antennas, (**b**) is the intermediate frequency signal sampler, and (**c**) is ProPak6 receiver.

To confirm that the new approach exhibits better behavior, real data tests were conducted. The comprehensive test platform is shown in Figure 14. It should be noted that only the GNSS antennas, the intermediate frequency signal sampler, and the ProPak6 receiver were used, the rest of the devices in the figure were used for other needs, and there will be no description here. The intermediate frequency signal sampler can convert the high frequency BDS B1 signal into a 3.996875 MHz digital IF signal with a sampling frequency of 16.369 MHz. The ProPak6 receiver is the most advanced receiver made by NovAtel Company seated in Calgary, Alberta, Canada, and the supplier is Beijing BDStar Navigation Co., Ltd. The ProPak6 receiver can provide meter to centimeter level positioning precision, thus it was used to judge the frequency accuracy of the algorithms. 

**Figure 15:**
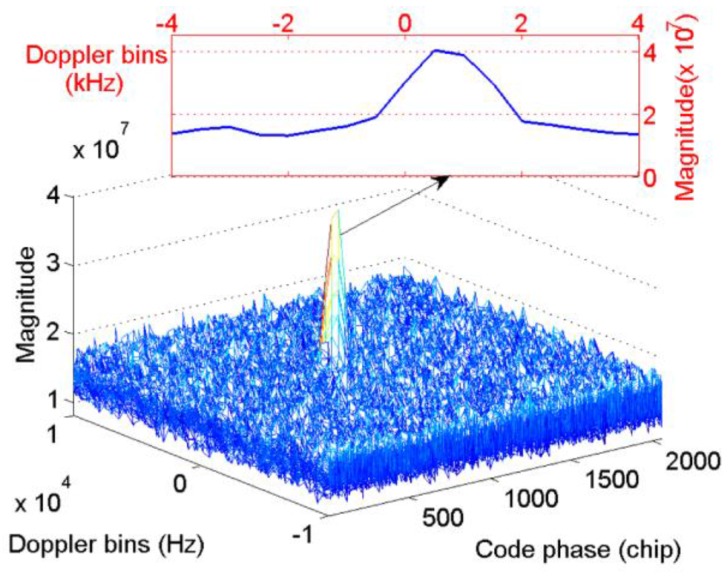
Real data test results of the conventional NC algorithm.

**Figure 16:**
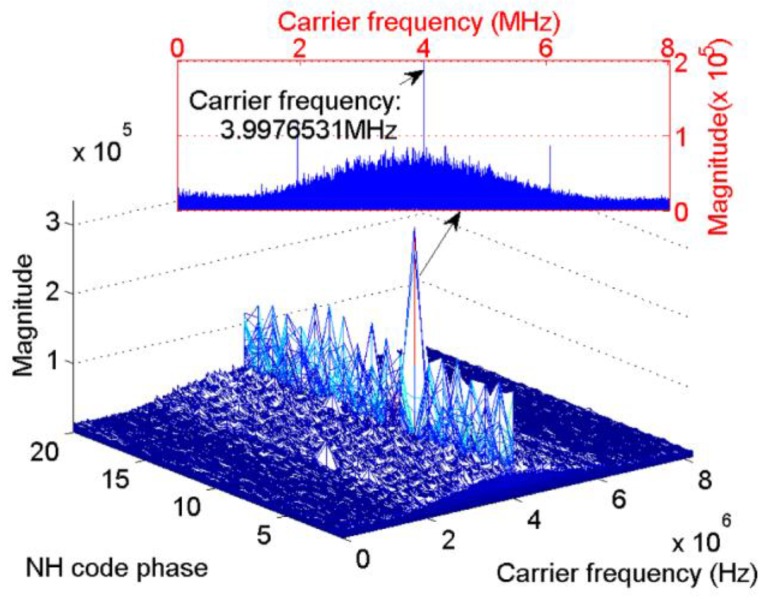
Real data test results of the proposed algorithm.

The parameters are as follows: the non-coherent integration time is 100 ms combined with 1 ms coherent integration, the data length of the proposed algorithm is 20 ms. Satellite 9 was visible and the Doppler bins obtained by the ProPak6 receiver is 739.355 Hz. The results of the conventional NC algorithm and the proposed scheme are shown in Figure 15 and Figure 16, respectively.

Figure 15 shows a slight peak split in the Doppler frequency axis caused by bit transitions, which results in a frequency error of 239.355 Hz compared with the reference receiver. The peak split is not serious because several integration results without bit transitions occur as a result of the long integration time. Further increasing the non-coherent integration time will hardly help mitigating the undesirable effect of bit transitions because the ratio of the results with, and without, bit transitions are determined by the structure of the NH codes. While Figure 16 mitigates this phenomenon by using the proposed algorithm, and shows that the start point of NH codes is eight, the frequency error is about 38.7 Hz compared with the reference Doppler, much smaller than the conventional one.

## 6. Conclusions

This study selects BDS signal as the research object. It addresses the acquisition of a weak signal channel, and derives the formula of CAF affected by bit transitions of conventional acquisition methods. The results indicate that the frequent bit transitions in BDS signals can cause a large frequency error up to 1000 Hz, which seriously affects the acquisition performance. To address this issue, a frequency recalculation algorithm is proposed. This algorithm uses an additional FFT operation based on the conventional NC algorithm and the coarse code phase, and can obtain the starting point of NH codes at the same time. Then, a fine DC acquisition process is performed to further correct the coarse code phase obtained from the conventional NC algorithm.

Simulations show that the new acquisition technique is reliable when the conventional NC is successful, and the new acquisition scheme exhibits excellent performance in terms of frequency accuracy and detection probability compared with conventional algorithms. More specifically, the proposed acquisition algorithm can acquire BDS signal of 30.3 dB-Hz effectively within 100 ms even if there exists frequent bit transitions, about 22% higher than conventional algorithms, meanwhile, the acquisition accuracy is higher than 20 Hz. When the delay of PRN codes is 0.5 chips in 45 dB-Hz, the detection probabilities of conventional acquisition algorithms is lower than 60%, while the new algorithm can reach 98% with an accuracy of about 25 Hz. In addition, the start point of NH codes can also be obtained, which is capable of long differential integration time. With the promotion of GNSS modernization, there will be a high demand of the signal acquisition algorithms.
